# Stricture and Retention of Urine

**Published:** 1823-04

**Authors:** Robert Rowley


ART. VI.—*Stricture and Retention of Urine.* By ROBERT ROWLEY, jun. Esq.

I OBSERVE, in your Number for December, the report of a case of retention of urine, wherein my name is mentioned; and my reason for now addressing you is, that I think its merits have been misunderstood by one of your readers, the Editor of a popular medical Journal, if I may judge from the remarks he has published on the subject.

It is true this was a most severe case of spasmodic stricture, resisting the usual methods of relief, but it furnished nothing new on that account; still I conceive it to be a case highly interesting to the profession, insomuch that it clearly demonstrates the benefit resulting from sangui-suction in one of the most obstinate, dangerous, and often most quickly fatal, of all diseases.

The introduction of an instrument by the adoption of such force as could safely be employed, was found utterly impracticable; for, although many surgeons will endeavour to evacuate the bladder at any rate by the *forcible* introduction of the catheter, yet it is a measure invariably attended with great danger to the patient; for, were an opportunity afforded to examine the seat of spasmodic stricture, the urethra, at the seat of the spasm, would be found so much contracted as scarcely to admit the passage of a bristle; but, when the spasm has subsided, the calibre of that canal is not diminished in the least, and therefore this species of stricture does not require dilatation, as in those of the permanent kind, but simply those means to allay spasm which were adopted in Dr. Price's case.

Numerous have been the cases of spasmodic stricture within my knowledge, where the forcible use of bougies and catheters had been employed for their relief, without any other means; but, in the majority of these, false passages were produced, and in not a few instances, a high degree of inflammation, mortification, and death: whereas I have frequently known cases, after the abstraction of blood and free use of opium, to yield so much that the introduction of a catheter could be effected merely by its own weight.

General blood-letting, to the extent of twenty-four ounces, had, however, been employed in this case without any benefit; purgatives, opiates, and the warm bath, had also been unsuccessfully administered; but, in about an hour after the copious evacuation of blood immediately from the seat of the stricture, by the application of eight leeches, the spasm ceased,—the urine flowed freely,—the catheter was afterwards introduced without difficulty,—and the patient has remained quite free from any symptoms of stricture ever since.

The beneficial effects resulting from the application of leeches to the perineum in cases of spasmodic stricture, would not however surprise us, were we to reflect for a moment on the real nature of its exciting cause—increased vascular action: therefore, unloading the blood-vessels of the affected part, by drawing blood from them directly through the medium of the anastamosing branches, must ever be the surest way of subduing the excitement and irritability thus set up. But these effects of sangui-suction in stricture, and a variety of other inflammatory and spasmodic affections, are so satisfactorily pointed out in a work recently published,* as to render any further observations by me unnecessary. I may however remark, that, notwith-

* *A Treatise on the Utility of Sangui-Suction, or Leech-Bleeding, in the Cure of various Diseases, &c.* By Rees Price, M.D. Surgeon.—12mo. London, 1822.

standing the Editor alluded to appears not to consider sangui-suction a *novel* remedy in spasmodic stricture, yet it cannot have been a very general one, never having known it adopted in the hospitals in the Borough, during the seven years that I was a student and dresser at St. Thomas's, at the Parisian hospitals, or even in private practice, until the publication of Dr. Price's work. Whether, however, it be a new practice or an old one revived, it matters not: I consider the profession equally indebted to the author for elucidating, in the perspicuous manner he has done, its importance in affections of the generative organs, and in diseases where the use of leech-bleeding had not been so satisfactorily explained.

Since the publication of the work alluded to, I have tried sangui-suction, in the manner recommended by Dr. Price, in several cases of *permanent* stricture, with the most decided advantage. One was that of a chemist and druggist residing in London, who, having to forward some copies of the London Medical and Physical Journal to his medical friends in the country, accidentally glanced his eye on Dr. Price's case of Retention of Urine. This gentleman had been afflicted with a stricture nine years, accompanied with exquisite pain and inconvenience in voiding his urine, and on the introduction of a bougie; which, by the by, could not be effected without great difficulty. He had likewise not unfrequently suffered much pain from complete retention of urine. The perusal of this case excited in him a wish to try the effects of leeching; but, prior to adopting it, he determined to take my opinion on the propriety of the measure, and upon his case generally. In attempting to pass a small sound, I discovered a projection situated on the upper surface of the urethra, immediately under the arch of the pubis, occasioned originally by an abscess in a lacuna: it projected so much into the urethra, as to render it necessary, in passing the sound, to keep the point downwards against the lower surface of the urethra, to enable it to pass.

Finding great difficulty in the introduction of the sound, and that excessive irritability and pain attended its use, I advised the application of a dozen leeches to the perineum, immediately over the stricture. These bled so profusely, that the patient assured me not less than a pound and half of blood had been evacuated, to the complete relief of those symptoms of irritation and pain which he had before experienced; and, by its removing the contractile force of the urethra, he was enabled to void his urine more freely. From this time I was able to pass daily an instrument of large size, without difficulty or pain; and, by a steady perseverance in the measure, with the combined use of medicines calculated to diminish the excitement of the part, he is now nearly cured.

83, *St. Margaret's Hill, Southwark;* 17*th January*, 1823.

